# Radiographic assessment of segmental correction and subsidence following posterior lumbar fusion with expandable interbody cages: a 12-month retrospective cohort study

**DOI:** 10.1007/s10143-025-03943-x

**Published:** 2025-11-29

**Authors:** David A. Hsiou, Hania Shahzad, Yashar Javidan

**Affiliations:** 1 St. Joseph’s Medical Center, Stockton, CA USA; 2 UC Davis Health, Sacramento, CA USA; 3 Department of Orthopaedic Surgery, UC Davis Medical Center, Sacramento, CA USA

**Keywords:** Expandable cages, Lumbar fusion, Segmental correction, Disc height, Subsidence

## Abstract

Expandable interbody cages have gained popularity in posterior lumbar fusion due to their ability to restore disc height and segmental alignment with minimal surgical exposure. However, concerns remain regarding their long-term durability, particularly the risk of implant subsidence. This study evaluates radiographic outcomes and subsidence rates over a 12-month period following posterior lumbar fusion with a novel expandable cage. We conducted a retrospective cohort study of 40 consecutive adult patients who underwent posterior lumbar fusion using a posteriorly placed expandable cage (Calibrate PSX) at a single academic institution. Radiographic parameters—including anterior disc height, posterior disc height, segmental lordosis, and global lumbar lordosis (L1–S1)—were measured preoperatively, immediately postoperatively, and at 3 and 12 months. Subsidence was graded according to the classification proposed by Levy et al. (2024). Generalized estimating equations were used for longitudinal analysis, with statistical significance set at *p* < 0.05. Among 44 implanted levels, 16 levels (36.4%) demonstrated subsidence at 3 months, increasing to 20 (45.5%) by 12 months. No surgical revisions were required at 12 months. Anterior disc height increased significantly in the immediate postoperative period (+ 8.6 mm, *p* < 0.0001) but declined over time, with greater loss in the subsidence group (–7.1 mm vs. − 1.9 mm, *p* = 0.013). Posterior disc height followed a similar trend. However, segmental and global lumbar lordosis remained stable, with no significant differences between subsidence and non-subsidence groups. Minor intraoperative complications occurred in 6 patients (13.6%) and resolved without sequelae. Expandable interbody cages provide substantial immediate postoperative improvements in disc height and alignment. While subsidence can occur over time, segmental and global lordosis are generally maintained. The overall subsidence rate was modest and not associated with adverse clinical outcomes. These findings support the cautious use of expandable cages in posterior lumbar fusion, with attention to patient selection, surgical technique and implant selection to mitigate the risk of subsidence.

## Introduction

Posterior lumbar interbody fusion (PLIF) and transforaminal lumbar interbody fusion (TLIF) are well-established surgical techniques used to treat a wide range of spinal pathologies, from simple degenerative disorders to complex deformities [1, 2]. While effective in achieving neural decompression and segmental stabilization, both PLIF and TLIF are limited in their ability to restore disc height and segmental lordosis. These limitations are largely attributed to the posterior approach, which restricts access to the intervertebral space and thereby limits the size and angulation of implants compared to more invasive anterior fusion techniques [3, 4]. Multiple meta-analyses have consistently demonstrated that posterior-based techniques utilizing static cages consistently perform below anterior-based techniques in restoration of segmental lordosis, global lumbar lordosis and disc height [5, 6].

To overcome these constraints, expandable interbody cages were introduced in the early 2000 s [7]. These devices are introduced in a collapsed state and then expanded in situ, allowing for greater disc height restoration and improved segmental alignment without requiring wider surgical exposure [8–10]. Over the past two decades, expandable cages have been found to confer biomechanical stability comparable to static cages [11, 12]. Careful patient selection remains critical, as expandable cages may be suboptimal in patients with poor-quality bone or requiring multilevel instrumentation [13, 14].

However, while theoretical advantages of expandable cages have been supported by improvements in intraoperative radiographic outcomes, questions remain regarding their mechanical durability and mid-to-long term performance. Cage subsidence, the collapse of interbody fusion cages into adjacent vertebral bodies, is an often-cited radiographic concern, though not always correlative with clinical symptoms [15]. In particular, several studies have reported higher rates of cage subsidence and loss of sagittal correction when compared to static devices, raising concerns about the ability of expandable implants to maintain intraoperatively restored height correction [16–18]. Conversely, a 2024 comparative study of TLIF expandable versus static cages demonstrated sustained radiographic correction, as well as superior patient-reported outcomes in the expandable cage cohort [19]. Subsidence following the use of expandable cages is multifactorial, with prior studies suggesting that excessive endplate stress during cage expansion and limited cage footprint may contribute to early settling [20, 21]. In patients with osteoporosis, studies have demonstrated that assessment of intraoperative overexpansion into poor-quality bone can be challenging [13]. In terms of patient-reported outcomes, recent studies have demonstrated comparable reductions in Oswestry Disability Index (ODI) and visual analog pain scores (VAS) between static cage and expandable cage implantation [19, 21].

Given the increasing adoption of expandable cages in posterior lumbar fusion and the need to better understand their long-term biomechanical stability, this study aims to evaluate radiographic outcomes following the use of a novel expandable interbody device. Specifically, we assess changes in disc height, segmental and lumbar lordosis, and the rate of implant subsidence over a 12-month postoperative period. We hypothesize that the use of a posterior expandable interbody device will result in significant immediate postoperative improvements in disc height and segmental alignment, but that a proportion of these gains will diminish over time due to implant subsidence.

## Materials and methods

### Study population

A retrospective, non-comparative, non-randomized observational study was conducted at a single academic center. Ethical approval was obtained prior to data collection, and all patients provided informed consent permitting the use of their de-identified clinical and radiographic data for research purposes. All adult patients (aged ≥ 18 years) between June 2022 to August 2023 who underwent posterior lumbar fusion with at least one level of a novel expandable interbody device (Calibrate PSX™ Interbody System, Alphatec Spine, Inc., Carlsbad, CA) were included in the study group. Sample size was determined via a convenience consecutive sampling method. Patients were excluded if they had severe osteoporosis (T < 2.5 + fragility fracture), uncontrolled systemic diseases that could impair bone healing, or incomplete radiographic follow-up data at the designated time points [22].

### Outcomes

The primary outcomes measured in this study were subsidence and cage failure, assessed through radiographic and clinical criteria over a 12-month follow-up period. Subsidence was evaluated according to the classification proposed by Levy et al. (2024) and categorized as follows [23]:

- Mild: < 2 mm loss in disc height
- Moderate: 2–4 mm loss
- Severe: >4 mm loss

Cage failure was defined as the presence of any of the following, irrespective of subsidence:

- Segmental lordosis loss > 5°,
- Reduction in disc height > 4 mm not attributable to measurable subsidence, or
- Cage migration > 3 mm in the anterior or posterior direction, as assessed on serial imaging.

Subsidence and failure analysis was determined by comparing intervertebral disc height on immediate postoperative and 3-month or 12-month radiographs. Disc height was calculated as the average of the anterior and posterior intervertebral space.

The secondary exploratory outcomes included the radiographic maintenance of segmental lordosis, anterior disc height, posterior disc height, and global lumbar lordosis (L1–S1) at 3 and 12 months postoperatively, in comparison to the immediate postoperative measurements. Additionally, the incidence of perioperative complications, reoperations, and device-related adverse events were recorded.

Radiographic measurements were obtained using standardized protocols on the Visage 7 software (Pro Medicus Ltd., Windows version). All imaging analyses were performed by an independent, trained investigator who was not involved in study design, surgical procedures, or patient follow-up, ensuring blinded and unbiased assessment.

### Surgical technique

All procedures were performed under general endotracheal anesthesia with the patient positioned prone on a radiolucent spinal table. A midline posterior incision was made over the affected lumbar segment after confirmation under fluoroscopy, followed by subperiosteal dissection of the paraspinal musculature to expose the posterior elements. A laminectomy was performed at the operative level, in conjunction with bilateral facetectomies to mobilize the segment. A thorough discectomy was performed using pituitary rongeurs. Interbody disc shavers were carefully used at 1 mm increments to remove disc material to the level of subchondral bone. Once sufficient counter resistance was felt upon rotation of the shaver and the position confirmed using lateral fluoroscopy, the corresponding height of the starting implant was selected. The Calibrate PSX device is available in widths of 10, 12 and 14 mm; lengths of 25 and 30 mm with starting heights of 7–12 mm in 1 mm increments and a maximum lordosis of 20 degrees. The selected implanted was delivered into the disc space in the collapsed state and then expanded using a torque limiting expansion driver under lateral fluoroscopy to restore disc height and lordosis. There are two torque handles provided in the instrument set with different torque thresholds: 13 in-lb and 20 in-lb. The selection of the torque limiting handle was not controlled in this study and was selected as per the surgeon’s discretion.

For two-level procedures, the steps above were repeated at the adjacent level. After cage expansion and confirmation of final position, pedicle screws were placed bilaterally. Pre-contoured rods were then secured to the screws to restore stability. Final fluoroscopy was used to confirm implant placement, alignment, and restoration of intervertebral height. Primary layered closure was performed in all cases.

### Statistical analysis

All data were collected in a structured spreadsheet and analyzed using R statistical software (version 4.2). Continuous variables were summarized using mean, median, standard deviation, interquartile range (IQR), and range. Categorical variables were described using absolute and relative frequencies.

For outcomes in which no events were observed, the Clopper–Pearson exact method was used to calculate the 95% confidence interval (CI) for the true event rate. This method, based on the binomial distribution, provides an exact interval that does not rely on large-sample approximations and is particularly appropriate for small sample sizes or rare events. The resulting interval represents the range within which the true proportion is expected to lie with 95% confidence, even when the observed event rate is zero.

Longitudinal changes in secondary outcomes were assessed using generalized estimating equations (GEE) with a Gaussian distribution and an autoregressive (AR-1) working correlation structure to account for within-subject correlations due to repeated measures over time.

Segmental lordosis outcomes were analyzed at the device level, treating each unique PSX device as the unit of analysis, while patient-level analyses were used for global lumbar lordosis (L1–S1) measurements. Estimated marginal means (EMMs) and their contrasts were calculated using the emmeans package. To improve the robustness of statistical inference in the presence of possible model misspecification, heteroscedasticity, or small sample bias, a sandwich-type variance estimator was applied. In particular, robust standard errors were obtained using the jackknife variance estimation method implemented in the clubSandwich package, which provides small-sample adjustments appropriate for clustered or longitudinal data structures. Pairwise comparisons of EMMs were corrected for multiple testing using the Benjamini–Yekutieli procedure, which accounts for possible dependencies among comparisons. A two-sided p-value < 0.05 was considered statistically significant.

## Results

### Patient demographics

A total of 55 consecutive patients were identified as potentially eligible in this study. However, after examination of medical records, 15 patients were excluded for incomplete imaging or loss to follow up. A total of 40 patients were included in the study, with a mean age of 66.4 years (range: 29–81 years). The cohort consisted of 50% males (*n* = 20) and 50% females (*n* = 20). The median number of comorbidities was 3 [IQR: 1–4], with a mean of 2.9 (± 2.4). Detailed demographic characteristics are summarized in Table 1.

**Table 1:** Demographic data of the sample. Med: Median; IQR: interquartile Range; std: standard Deviation; N: number of observations; NA: missing or not applicable

Variable	Statistics/Items	Value
Age (Years)	Min/Max	29.0/81.0
	**Med [IQR]**	**70.5 [59.0;75.2]**
	Mean (std)	66.4 (11.0)
	N (NA)	40 (0)
Gender	Female	20 (50.00%)
	Male	20 (50.00%)
Comorbidities (Count)	Min/Max	0/12.0
	**Med [IQR]**	**3.0 [1.0;4.0]**
	Mean (std)	2.9 (2.4)
	N (NA)	40 (0)

### Surgical characteristics

Among the 40 patients, 36 (81.8%) underwent single-level implantation of the expandable posterior interbody device (PSX), while 8 patients (18.2%) received two-level implants, totaling 44 operated levels. The most frequently treated level was L5–S1 (40.9%), followed by L4–L5 (31.8%). All implanted levels were stabilized with pedicle screw fixation, with a median instrumentation length of two levels [IQR: 1–4]. The majority of the cages used had a posterior height of either 7 mm (40.9%) or 8 mm (43.2%), a width of 10 mm (79.6%), and a length of 25 mm (88.6%). Intraoperative complications were observed in 6 patients (13.6%), primarily involving technical adjustments without adverse clinical outcomes. Additional data, including implant dimensions, are detailed in Table 2.

**Table 2:** Surgical data. Med: Median; IQR: interquartile Range; std: standard Deviation; N: number of observations; NA: missing or not applicable

Variable	Statistics/Items	Value
Operated Levels with PSX (Number)	1	36 (81.82%)
	2	8 (18.18%)
Operated Levels with PSX (Vertebral Level)	L2L3	6 (13.64%)
	L3L4	6 (13.64%)
	L4L5	14 (31.82%)
	L5S1	18 (40.91%)
Total Construct Posterior Instrumentation Levels (Number)	Min/Max	1.0/14.0
	Med [IQR]	2.0 [1.0;4.0]
	Mean (std)	3.5 (3.3)
	N (NA)	44 (0)
Total Construct Length (Upper Instrumented Vertebra – Lower Instrumented Vertebra)	L2-L3	1 (2.27%)
	L2-L5	2 (4.55%)
	L2-S1	3 (6.82%)
	L3-L4	1 (2.27%)
	L3-L5	3 (6.82%)
	L3-S1	5 (11.36%)
	L4-L5	8 (18.18%)
	L4-S1	6 (13.64%)
	L5-S1	5 (11.36%)
	T10-S1	2 (4.55%)
	T11-L3	1 (2.27%)
	T11-S1	3 (6.82%)
	T4-S1	2 (4.55%)
	T9-S1	2 (4.55%)
Cage Height (mm)	7	18 (40.91%)
	8	19 (43.18%)
	10	7 (15.91%)
Cage Width (mm)	10	35 (79.55%)
	14	9 (20.45%)
Cage Length (mm)	25	39 (88.64%)
	30	5 (11.36%)
Intraoperative Complications (Per Patient)	No	34 (86.36%)
	Yes	6 (13.64%)

### Subsidence and implant performance

At the 3-month follow-up, moderate subsidence was identified in 14 levels (30%) and severe subsidence was identified in 2 levels (4%). By the 12-month follow-up, 14 levels (30%) demonstrated moderate subsidence and 6 levels (13%) presented severe subsidence (Table 3). No cases met clinical or radiographic significance to require surgical revision during the follow-up period. No cases of cage failure (e.g., migration, collapse, or reoperation) were observed in the cohort of 44 patients (0/44). In addition, analysis of follow-up radiographs did not demonstrate any signs of pedicle screw loosening, despite evidence of subsidence.

**Table 3:** Subsidence rate at 3 and 12 months. The subsidence rate was defined as: no subsidence < 2 mm, moderate > 2 mm disc Heigh loss, severe > 4 mm disc Height loss

3 months		
	N	%
No Subsidence	28	62
Moderate Subsidence	14	30
Severe Subsidence	2	8
12 Months		
	N	%
No Subsidence	24	55
Moderate Subsidence	14	32
Severe Subsidence	6	13

### Anterior disc height

There was a statistically significant increase in anterior disc height from the preoperative to the immediate postoperative period (mean increase: 8.6 mm; 95% CI: 5.9–11.4; *p* < 0.0001). However, this was followed by a significant reduction at 3 months (–3.0 mm; 95% CI: − 4.4 to − 1.8; *p* < 0.0001) and at 12 months (–4.5 mm; 95% CI: − 6.5 to − 2.5; *p* < 0.0001). Specific data are presented in Table 4.

### Posterior disc height

Posterior disc height also increased significantly postoperatively (mean increase: 6.7 mm; 95% CI: 4.2–8.1; *p* < 0.0001) but declined over time. A significant decrease was noted at both 3 months (–1.6 mm; 95% CI: − 3.0 to − 0.4; *p* < 0.0001) and 12 months (–2.5 mm; 95% CI: − 4.0 to − 1.1; *p* < 0.0001). Specific data are presented in Table 4.

**Table 4:** Anterior and posterior disc height across follow-up (FUP) time points. Values are reported as minimum/maximum, median [interquartile range, IQR], and mean (standard deviation, SD); N (NA) indicates the number of observations and missing values at each time point. Measurements are shown for preoperative, immediate postoperative, 1–3 months, and 12 months. Values in millimeters unless otherwise specified. Abbreviations: FUP, follow-up; IQR, interquartile range; SD, standard deviation; NA, not available

	Statistics	FUP			
		Preoperative	Immediate Postop	1–3 Months	12 Months
Anterior Disc Height	Min/Max	3.2/17.8	11.3/28.0	9.7/23.6	9.2/21.8
	Med [IQR]	8.4 [6.8;10.6]	17.3 [15.2;18.8]	14.8 [12.6;17.5]	14.6 [12.6;16.8]
	Mean (std)	9.0 (3.5)	17.4 (3.6)	15.3 (3.5)	14.6 (2.8)
	N (NA)	44 (0)	44 (0)	44 (0)	44 (0)
Posterior Disc Height	Min/Max	1.6/10.7	5.3/19.0	5.0/14.5	5.1/13.1
	Med [IQR]	4.7 [3.2;5.8]	9.4 [8.4;10.3]	8.3 [7.3;9.4]	8.1 [7.2;9.5]
	Mean (std)	4.7 (1.8)	9.8 (2.6)	8.8 (2.1)	8.4 (1.8)
	N (NA)	44 (0)	44 (0)	44 (0)	44 (0)

### Segmental lordosis

There were statistically significant differences in segmental lordosis between the preoperative and immediate postoperative (mean increase: 4.8°, 95% CI [−8.16;−1.45], *p* = 0.001), 3-month postoperative (mean increase: 5.1°, 95% CI [−8.83;−1.55], *p* = 0.001), and 12-month postoperative (mean increase: 4.9°, 95% CI [−8.28;−1.59], *p* = 0.001) periods.

### Lumbar lordosis (L1–S1)

Global lumbar lordosis (L1–S1) remained stable across all time points. No significant changes were observed from the postoperative period to either the 3- or 12-month follow-ups in either the subsidence or non-subsidence group.

## Discussion

### Strengths and limitations

This study possesses several methodological strengths. Notably, it provides a comprehensive, time-resolved radiographic assessment of anterior and posterior disc height, segmental lordosis, and global lumbar alignment over a 12-month period following posterior lumbar fusion, among consecutive patients. Imaging measurements were preemptively standardized and performed by an independent researcher. Several limitations should be discussed. The retrospective and single-center nature of the study does inherently introduce selection bias and limits the ability to control for confounding variables, such as implant positioning and surgical technique variations. Additionally, the absence of a comparator group utilizing static cages precludes direct evaluation of the relative performance of expandable versus non-expandable devices. Finally, although a 12-month follow-up period provides valuable insight into early device behavior, it may be insufficient to fully characterize long-term implant performance and the potential for delayed subsidence or adjacent segment pathology. This study did not standardize post-operative imaging with full length standing films which may affect global lumbar lordosis results due to unseen compensatory mechanisms.

### Implications and future actions

Expandable interbody cages represent a relatively recent innovation in spinal fusion, offering a customized approach to restoring disc height and segmental alignment. While current literature supports the utility of expandable cages in achieving significant postoperative improvements in both anterior and posterior disc height, as well as segmental lordosis [24, 25], concerns persist regarding long-term stability, particularly due to subsidence. This study aimed to evaluate whether posterior expandable cages could maintain disc height and lordosis correction over time. As hypothesized, we found a significant postoperative increase in both anterior and posterior disc height, as well as segmental lordosis. However, a portion of this correction was lost by 12 months, primarily in cases with documented subsidence. Importantly, while disc height loss was significantly greater in the subsidence group, segmental and lumbar lordosis remained largely preserved, suggesting that these devices may sustain sagittal alignment even when subsidence occurs.

Our results complement those reported by Kucharzyk et al. (2023), which demonstrated greater disc height and segmental lordosis correction with expandable cages compared to static alternatives [26]. Similarly, Lin et al. (2022) conducted a meta-analysis revealing that expandable cages provided superior anterior disc and foraminal height restoration, though no significant differences in segmental lordosis were found compared to static cages [27]. These findings are echoed in our study, where disc height was significantly improved postoperatively but subject to a degree of loss over time.

Several studies have cautioned that the benefits of expandable cages may be offset by their higher susceptibility to subsidence. Chang et al. (2020) reported a subsidence rate of 19% for expandable devices compared to 5% for static devices, with particularly high rates observed in cases involving minimal facet resection [28]. Armocida et al. (2021) similarly noted a higher rate of subsidence with expandable cages (22% vs. 8%), which ultimately resulted in no significant improvement in disc height or lordosis at final follow-up [17]. More recently, Crawford et al. (2025) found subsidence rates of 14.4% for expandable devices versus 6.6% for static cages [16]. These findings are consistent with the present study, where a modest rate of subsidence (15.9%) was observed, and where cases of subsidence were associated with greater loss of correction, particularly in anterior and posterior disc height.

Notably, while axial correction was subject to subsidence over time, segmental and global lordosis remained stable upon analysis of serial radiographs over the 12-month period. This preservation of sagittal plane correction is reasonably attributed to proportional losses in anterior disc height and posterior disc height [29, 30]. Symmetric subsidence would incur minimal changes to lordosis over time. Furthermore, other components of the surgical construct, including precontoured rods, can support lordotic correction [28]. Particularly in multilevel posteriorly-instrumented constructs, the effect of subsidence on global lordosis over time may be negligible.

Overall, while expandable cages offer immediate anatomical restoration, their long-term mechanical durability remains a critical area of concern. Our findings underscore the importance of careful patient selection, surgical technique, and implant sizing to minimize the risk of subsidence. Future studies which control the use of the torque limiting handles with matched cohorts would be of interest. Future studies with larger cohorts and prospective designs are needed to further evaluate the clinical implications of subsidence and to optimize the use of expandable technologies in posterior lumbar fusion. Additional studies evaluating long term outcomes beyond 12 months may help complete understanding of the effects of identified subsidence on overall clinical outcomes. In addition, although this cohort did not demonstrate pedicle screw loosening in the context of subsidence on serial radiographs, future studies can better characterize possible presence of loosening via computed tomography. Finally, postoperative fusion rates were not investigated, which may be of clinical interest and should be studied further in future cohorts.

## Conclusions

The present study suggests that expandable cages can promote a significant increase in disc height (both anterior and posterior); however, partial loss of both anterior and posterior disc height should be expected during follow-up. Importantly, segmental and global lumbar lordosis remained stable in the follow up period, indicating that expandable implants demonstrate reliable maintenance of spinal sagittal balance.

## Data Availability

No datasets were generated or analysed during the current study.
